# Dual Synchronization of Fractional-Order Chaotic Systems via a Linear Controller

**DOI:** 10.1155/2013/159194

**Published:** 2013-09-14

**Authors:** Jian Xiao, Zhen-zhen Ma, Ye-hong Yang

**Affiliations:** College of Mathematics and Statistics, Chongqing University, Chongqing 401331, China

## Abstract

The problem of the dual synchronization of two different fractional-order chaotic systems is studied. By a linear controller, we realize the dual synchronization of fractional-order chaotic systems. Finally, the proposed method is applied for dual synchronization of Van der Pol-Willis systems and Van der Pol-Duffing systems. The numerical simulation shows the accuracy of the theory.

## 1. Introduction

In recent years, the topic of chaos synchronization has attracted increasing attention in many fields. The result of synchronization of chaotic oscillators is used in nonlinear oscillators [1], circuit experiment [2], secret communication [3], and some other fields. In 1990, the first concept of synchronization was presented by Carroll and Perora [4]. And there are many methods about chaos synchronization such as Lyapunov equation [5], Perora-Carroll (PC) [4] and backstepping control [6]. All of these methods are amid of the synchronization between one master and one slave system do not consist of the synchronization of multimaster systems and multislave systems.

Dual synchronization is a special circumstance in synchronization of chaotic oscillators. The first idea of multiplexing chaos using synchronization was investigated in a small map and an electronic circuit model by Tsimring and Sushchik in 1996 in [7]; then the concept of dual synchronization was raised by Liu and Davids in 2000 in [8], which concentrates on using a scalar signal to simultaneously synchronize two different pairs chaotic oscillators, that is, the synchronization between two master systems and two slave systems.

Nowadays, there are many dual synchronization methods, such as in 2000 Liu and Davids introduce the dual synchronization of 1-D discrete chaotic systems via specific classes of piecewise-linear maps with conditional linear coupling in [8]. The dual synchronization between the Lorenz and Rossler systems by the Lyapunov stabilization theory is investigated in [9]. The output feedback strategy is used to study the dual synchronization of two different 3-D continuous chaotic systems in [10]. Then the dual synchronization in modulated time-delayed systems is investigated by designing a delay feedback controller in [11]. All of these works are amid of the dual synchronization of integer-order chaotic systems and do not consist of the dual synchronization of fractional-order chaotic systems. In this paper, a new method of dual synchronization of fractional-order chaotic systems is proposed, by a linear controller; the dual synchronization of chaos is obtained. 

The rest of this paper is organized as follows: in Section 2, we construct a theory frame about the dual synchronization of two different fractional-order chaotic systems. By a linear controller, we obtain dual synchronization between two different fractional-order chaotic systems in Section 3. In Section 4, the proposed method is applied to dual synchronization of Van der Pol-Willis systems and Van der Pol-Duffing systems for evaluating the performance of the method and by numerical simulation; the result shows that the controller designed by the application of this method is effective. Finally, conclusions are drawn in Section 5. 

## 2. Problem Analysis

We define the following two systems as two master systems.

*Master 1:*

(1) $$\begin{array}{c} \frac{d^{\alpha }x}{dt^{\alpha }}=f\left(t,x\right), \end{array}$$

*Master 2:*

(2) $$\begin{array}{c} \frac{d^{\alpha }y}{dt^{\alpha }}=g\left(t,y\right), \end{array}$$

where *x* = [*x*_1_, *x*_2_,…, *x*_*n*_]^*T*^ and *y* = [*y*_1_, *y*_2_,…, *y*_*m*_]^*T*^ are the state vectors of the two master systems.   *f* ∈ *C*[*R* ^+^ × *R* ^*n*^,  *R* ^*n*^] and *g* ∈ *C*[*R* ^+^ × *R* ^*m*^,  *R* ^*m*^] are two known functions. *α* ∈ (0,1] is the order of the two master systems. By a linear combination of the two master systems states, a signal *v*_*m*_ is give as

(3) vm=∑i=nnaixi+∑j=1mbjyj=[a1,a2,…,an]x+[b1,b2,…,bm]y=Ax+By=[AB][xy]=CTξ,

where *A* = [*a*_1_, *a*_2_,…, *a*_*n*_]^*T*^ and *B* = [*b*_1_, *b*_2_,…, *b*_*m*_]^*T*^ are two known matrices, and *a*_*i*_, *b*_*j*_, *i* = 1,2,…, *n*, *j* = 1,2,…, *m* cannot be zero at the same time. So $P={\left[\begin{array}{cc} A^{T} & B^{T} \end{array}\right]}^{T}$ is a known matrix. $\xi ={\left[\begin{array}{cc} x^{T} & y^{T} \end{array}\right]}^{T}$ is a combination of the two master systems states. The corresponding two slave systems are as follows:

*Slave 1:*

(4) $$\begin{array}{c} \frac{d^{\alpha }X}{dt^{\alpha }}=f\left(t,X\right)+U^{\left(1\right)}, \end{array}$$

*Slave 2:*

(5) $$\begin{array}{c} \frac{d^{\alpha }Y}{dt^{\alpha }}=g\left(t,Y\right)+U^{\left(2\right)}, \end{array}$$

where *X* = [*X*_1_, *X*_2_,…, *X*_*n*_]^*T*^ and *Y* = [*Y*_1_, *Y*_2_,…, *Y*_*m*_]^*T*^ are the state vectors of the two slave systems, *U* ^(1)^ = [*u*_1_^1^, *u*_2_^1^,…, *u*_*n*_^1^]^*T*^ and *U* ^(2)^ = [*u*_1_^2^, *u*_2_^2^,…, *u*_*m*_^2^]^*T*^ are vectors of manipulated variables, and *α* ∈ (0,1] is the order of the two slave systems. Similarly, by a linear combination of two slave systems states, a signal *v*_*s*_ is generated as follows:

(6) vs=∑i=nnaiXi+∑j=1mbjYj=[a1,a2,…,an]X+[b1,b2,…,bm]Y=AX+BY=[AB][XY]=CTη,

where $\eta ={\left[\begin{array}{cc} X^{T} & Y^{T} \end{array}\right]}^{T}$ is a combination of the two slave systems states.

The error signal for dual synchronization is

(7) $$\begin{array}{c} e=v_{s}-v_{m}=\left[\begin{array}{cc} A & B \end{array}\right]\left[\begin{array}{c} X-x\\ Y-y \end{array}\right]=C^{T}\left(\eta -\xi \right). \end{array}$$

The main goal is to synchronize the master systems and the slave systems is equivalent to

(8) $$\begin{array}{c} \underset{t\to \infty }{\lim}||X\left(t\right)-x\left(t\right)||=0,\;\;\underset{t\to \infty }{\lim}||Y\left(t\right)-y\left(t\right)||=0, \end{array}$$

where ||·|| is the Euclidian norm.

## 3. Dual Synchronization Strategy

**Lemma 1**

Considering the fractional-order system

(9) $$\begin{array}{c} D^{\alpha }z\left(t\right)=Qz,\;\;\;z\left(0\right)=z_{0}, \end{array}$$

where 0 < *α* ≤ 1, *z* ∈ *R* ^*n*^, and *Q* ∈ *R* ^*n*×*n*^; then system (9) is stable if and only if |arg(*λ*_*i*_(*Q*))| ≥ (*απ*/2), *i* = 1,2,…, where arg(*λ*_*i*_(*Q*)) denotes the argument of the eigenvalue *λ*_*i*_ of *Q*.

**Theorem 2**

The dual synchronization of fractional-order chaotic systems between the master systems and the slave systems is achieved if and only if the following condition satisfies

(10) $$\begin{array}{c} \left|\arg\left(\text{eig}\left(G\left(t\right)+KC^{T}\right)\right)\right|\geq \frac{\alpha \pi }{2}, \end{array}$$

where *G*(*t*) is the coefficient matrix of master systems and *K* is a control gain vector.

**Proof**

We can rewrite (1) and (2) in the following form by defining $\Psi ={\left[\begin{array}{cc} f & g \end{array}\right]}^{T}$:

(11) $$\begin{array}{c} \left[\begin{array}{c} \frac{d^{\alpha }x}{dt^{\alpha }}\\ \frac{d^{\alpha }y}{dt^{\alpha }} \end{array}\right]=\left[\begin{array}{c} f\left(t,x\right)\\ g\left(t,y\right) \end{array}\right],\;\;\frac{d^{\alpha }\xi }{dt^{\alpha }}=\Psi \left(t,\xi \right). \end{array}$$

Similarly, (4) and (5) can be rewritten as

(12) $$\begin{array}{c} \left[\begin{array}{c} \frac{d^{\alpha }X}{dt^{\alpha }}\\ \frac{d^{\alpha }Y}{dt^{\alpha }} \end{array}\right]=\left[\begin{array}{c} f\left(t,X\right)+U^{\left(1\right)}\\ g\left(t,Y\right)+U^{\left(2\right)} \end{array}\right],\;\;\frac{d^{\alpha }\eta }{dt^{\alpha }}=\Psi \left(t,\eta \right)+U, \end{array}$$

where *U* = [(*U* ^(1)^)^*T*^  (*U* ^(2)^)^*T*^]^*T*^, one defines $U=\left[\begin{array}{c} U^{\left(1\right)}\\ U^{\left(2\right)} \end{array}\right]=\left[\begin{array}{c} K_{1}e\\ K_{2}e \end{array}\right]$ and $E=\left[\begin{array}{c} X-x\\ Y-y \end{array}\right]$.

Equation (12) is transformed into

(13) $$\begin{array}{c} \left[\begin{array}{c} \frac{d^{\alpha }X}{dt^{\alpha }}\\ \frac{d^{\alpha }Y}{dt^{\alpha }} \end{array}\right]=\left[\begin{array}{c} f\left(t,X\right)+K_{1}e\\ g\left(t,Y\right)+K_{2}e \end{array}\right], \end{array}$$

so the error system is transformed into

(14) $$\begin{array}{c} \frac{d^{\alpha }E}{dt^{\alpha }}=\left[\begin{array}{c} \frac{d^{\alpha }X}{dt^{\alpha }}-\frac{d^{\alpha }x}{dt^{\alpha }}\\ \frac{d^{\alpha }Y}{dt^{\alpha }}-\frac{d^{\alpha }y}{dt^{\alpha }} \end{array}\right]=\frac{d^{\alpha }\eta }{dt^{\alpha }}-\frac{d^{\alpha }\xi }{dt^{\alpha }}. \end{array}$$

The error system is obtained as

(15)   dαηdtα−dαξdtα  =Ψ(t,η)+Ke−Ψ(t,ξ)=Ψ(t,η)−Ψ(t,ξ)+Ke  =Ψ(t,ξ+E)−Ψ(t,ξ)+K(η−ξ)  =Ψ(t,ξ+E)−Ψ(t,ξ)+KCTE.

Using the first-order Taylor expansion, the function Ψ(·) is rewritten as

(16) Ψ(t,ξ+E)−Ψ(t,ξ) =∂Ψ(t,ξ)∂ξE+h.o.t=G(t)E+h.o.t,

where h.o.t denotes the higher order terms of the series. We substitute (16) into (15) and yield

(17) dαEdtα=G(t)E+h.o.t+KCTE=[G(t)+KCT]E+h.o.t.

We can transfer the (17) into

(18) $$\begin{array}{c} \frac{d^{\alpha }E}{dt^{\alpha }}=\left[G\left(t\right)+KC^{T}\right]E \end{array}$$

according to Lemma 1, we can know that the error system is asymptotically stable at zero if and only if the following condition is satisfied

(19) $$\begin{array}{c} \left|\arg\left(\text{eig}\left(G\left(t\right)+KC^{T}\right)\right)\right|\geq \frac{\alpha \pi }{2}. \end{array}$$

## 4. The Example Analysis and Numerical Simulations

**Example 3 (dual synchronization of Van der Pol-Willis systems)**

In the first example, we can use the proposed method to achieve the dual synchronization of the Van der Pol system and the Willis system.

*Master 1:* Van der Pol system

(20) $$\begin{array}{c} \frac{d^{\alpha }x_{1}}{dt^{\alpha }}=x_{1}-\gamma x_{1}^{3}-\beta x_{2}+f_{1}\cos t,\\ \frac{d^{\alpha }x_{2}}{dt^{\alpha }}=l\left(x_{1}-mx_{2}+n\right). \end{array}$$

*Master 2:* Willis system

(21) $$\begin{array}{c} \frac{d^{\alpha }y_{1}}{dt^{\alpha }}=y_{2},\\ \frac{d^{\alpha }y_{2}}{dt^{\alpha }}=ay_{1}+by_{1}^{2}+cy_{1}^{3}+dy_{2}+f_{2}\cos t. \end{array}$$

So the corresponding slave systems are as follows:

*Slave 1:*

(22) $$\begin{array}{c} \frac{d^{\alpha }X_{1}}{dt^{\alpha }}=X_{1}-\gamma X_{1}^{3}-\beta X_{2}+f_{1}\cos t+k_{1}e,\\ \frac{d^{\alpha }X_{2}}{dt^{\alpha }}=l\left(X_{1}-mX_{2}+n\right)+k_{2}e, \end{array}$$

*Slave 2:*

(23) $$\begin{array}{c} \frac{d^{\alpha }Y_{1}}{dt^{\alpha }}=Y_{2}+k_{3}e,\\ \frac{d^{\alpha }Y_{2}}{dt^{\alpha }}=aY_{1}+bY_{1}^{2}+cY_{1}^{3}+dY_{2}+f_{2}\cos t+k_{4}e, \end{array}$$

where *e* = *a*_1_ *e*_1_ + *a*_2_ *e*_2_ + *b*_1_ *e*_3_ + *b*_2_ *e*_4_,  *e*_1_ = *X*_1_ − *x*_1_,  *e*_2_ = *X*_2_ − *x*_2_,  *e*_3_ = *Y*_1_ − *y*_1_, and *e*_4_ = *Y*_2_ − *y*_2_.

The *G*(*t*) matrix of the master systems is achieved as

(24) $$\begin{array}{c} G\left(t\right)=\left\lceil \begin{array}{cccc} 1-3\gamma x_{1}^{2} & -\beta & 0 & 0\\ l & lm & 0 & 0\\ 0 & 0 & 0 & 1\\ 0 & 0 & a+2by_{1}+3cy_{1}^{2} & d \end{array}\right\rceil , \end{array}$$

so the corresponding error matrix are as follows:

(25) (dαe1dtαdαe2dtαdαe3dtαdαe4dtα)=(1−3γx12+a1k1−β+a2k1b1k1b2k1l+a1k2lm+a2k2b1k2b2k2a1k3a2k3b1k31+b2k3a1k4a2k4a+2by1+3cy12+b1k4d+b2k4) ×(e1e2e3e4).

We should choose the appropriate parameters so that all the eigenvalues of the Jacobian matrix of (25) satisfy Matignon condition; that is, the eigenvalues evaluated at the equilibrium point are satisfied:

(26) $$\begin{array}{c} \left|\arg\left(\text{eig}\left(G\left(t\right)+KC^{T}\right)\right)\right|>\frac{\alpha \pi }{2}. \end{array}$$

The eigenvalue equation of the equilibrium point is locally asymptotically stable. From what we have discussed above, we can know that *A* and *B* are two known matrices; the parameter *K* can be appropriately selected for satisfying the Matignon condition. 

Dual synchronization of the Van der Pol system and the Willis system is simulated. The system parameters are set to be *γ* = 1/3, *β* = 1, *f*_1_ = 0.74, *l* = 0.1, *m* = 0.8, *n* = 0.7, *a* = −0.9, *b* = 3, *c* = −2, *d* = −0.1, *f*_2_ = 0.1, *A* = [1,1, 1], *B* = [1,1, 1],  and *α* = 1, so

(27) G(t)+KCT=(1−x12+k1−1+k1k1k10.1+k2−0.08+k2k2k2k3k3k31+k3k4k4−0.9+6y1−6y12+k4−0.1+k4).

**Figure 1:**
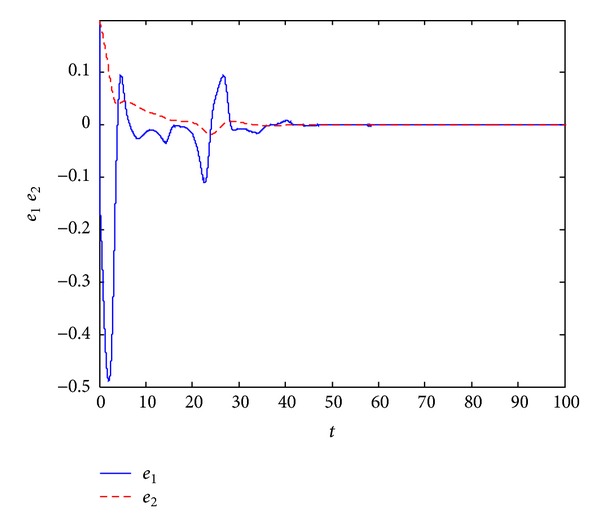
Error signals between the pair of Van der Pol system.

**Figure 2:**
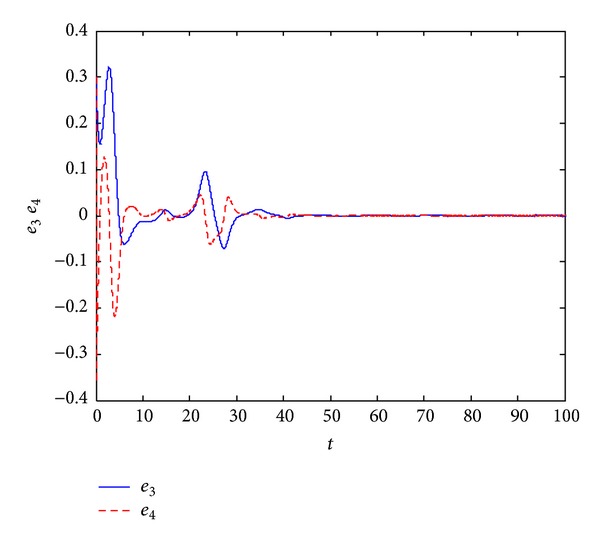
Error signals between the pair of Willis system.

If −295 < *k*_1_ < −130, *k*_2_ = −0.1, *k*_3_ = −1, and *k*_4_ = −400, which satisfy (18), the eigenvalue equation of the equilibrium point is locally asymptotically stable. We choose  *k*_1_ = −210, *k*_2_ = −0.1, *k*_3_ = −1, and *k*_4_ = −400. The initial conditions of the master system 1 and the master system 2 are taken as *x*_1_(0) = 0.1, *x*_2_(0) = 0.2 and *y*_1_(0) = 0.2,  *y*_2_(0) = 0.3; the initial conditions of the slave system 1 and the slave system 2 are taken as *X*_1_(0) = 0.3,  *X*_2_(0) = 0.4 and *Y*_1_(0) = 0.5, *Y*_2_(0) = 0.6, so the initial conditions of the error system are set to be *e*_1_(0) = 0.2, *e*_2_(0) = 0.2, *e*_3_(0) = 0.3, and *e*_4_(0) = 0.3. In Figures 1 and 2, we can see that all error variables have converged to zero; that is, we achieve the dual synchronization between the Van der Pol and the Willis systems.

**Example 4 (dual synchronization of Van der Pol and Duffing systems)**

For Example 4, the dual synchronization of Van der Pol and Duffing systems is investigated.

*Master 1:* Van der Pol system

(28) $$\begin{array}{c} \frac{d^{\alpha }x_{1}}{dt^{\alpha }}=x_{1}-\gamma x_{1}^{3}-\beta x_{2}+f_{1}\cos t,\\ \frac{d^{\alpha }x_{2}}{dt^{\alpha }}=l\left(x_{1}-mx_{2}+n\right). \end{array}$$

*Master 2:* Duffing system

(29) $$\begin{array}{c} \frac{d^{\alpha }y_{1}}{dt^{\alpha }}=y_{2},\\ \frac{d^{\alpha }y_{2}}{dt^{\alpha }}=ay_{1}+by_{1}^{3}+cy_{2}+f_{2}\cos t. \end{array}$$

So the corresponding slave systems are

*Slave 1:*

(30) $$\begin{array}{c} \frac{d^{\alpha }X_{1}}{dt^{\alpha }}=X_{1}-\gamma X_{1}^{3}-\beta X_{2}+f_{1}\cos t+k_{1}e,\\ \frac{d^{\alpha }X_{2}}{dt^{\alpha }}=l\left(X_{1}-mX_{2}+n\right)+k_{2}e, \end{array}$$

*Slave 2:*

(31) $$\begin{array}{c} \frac{d^{\alpha }Y_{1}}{dt^{\alpha }}=Y_{2}+k_{3}e,\\ \frac{d^{\alpha }Y_{2}}{dt^{\alpha }}=aY_{1}+bY_{1}^{3}+cY_{2}+f_{2}\cos t+k_{4}e, \end{array}$$

where *e* = *a*_1_ *e*_1_ + *a*_2_ *e*_2_ + *b*_1_ *e*_3_ + *b*_2_ *e*_4_, *e*_1_ = *X*_1_ − *x*_1_,  *e*_2_ = *X*_2_ − *x*_2_, *e*_3_ = *Y*_1_ − *y*_1_, and *e*_4_ = *Y*_2_ − *y*_2_.

The *G*(*t*) matrix of the master systems is achieved as

(32) $$\begin{array}{c} G\left(t\right)=\left\lceil \begin{array}{cccc} 1-3\gamma x_{1}^{2} & -\beta & 0 & 0\\ l & lm & 0 & 0\\ 0 & 0 & 0 & 1\\ 0 & 0 & a+3by_{1}^{2} & c \end{array}\right\rceil . \end{array}$$

So the corresponding error matrix are as follows:

(33) (dαe1dtαdαe2dtαdαe3dtαdαe4dtα)=(1−3γx12+a1k1−β+a2k1b1k1b2k1l+a1k2lm+a2k2b1k2b2k2a1k3a2k3b1k31+b2k3a1k4a2k4a+3by12+b1k4c+b2k4) ×(e1e2e3e4).

We should choose the appropriate parameters so that all the eigenvalues of the Jacobian matrix of (33) satisfy Matignon condition; that is, the eigenvalues evaluated at the equilibrium point are satisfied:

(34) $$\begin{array}{c} \left|\arg\left(\text{eig}\left(G\left(t\right)+KC^{T}\right)\right)\right|>\frac{\alpha \pi }{2}. \end{array}$$

The eigenvalue equation of the equilibrium point is locally asymptotically stable. Because *A* and *B* are two known matrices, the parameter *K* can be appropriately selected for satisfying the Matignon condition. 

According to what we have studied above, parameters are set to *γ* = 1/3, *β* = 1,  *f*_1_ = 0.74, *l* = 0.1, *m* = 0.8, *n* = 0.7, *a* = 1, *b* = −1, *c* = −0.15, *f*_2_ = 0.3, *A* = [1,1, 1], *B* = [1,1, 1], and *α* = 0.98, so

(35) G(t)+KCT=(1−x12+k1−1+k1k1k10.1+k2−0.08+k2k2k2k3k3k31+k3k4k41−3y12+k4−0.15+k4).

**Figure 3:**
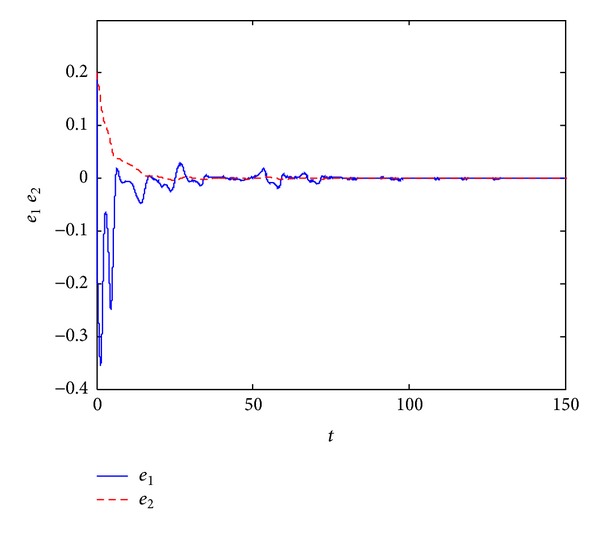
Error signals between the pair of Van der Pol system.

**Figure 4:**
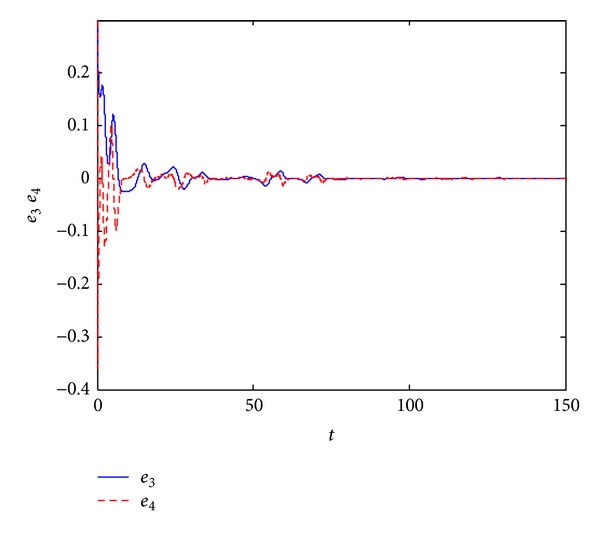
Error signals between the pair of Duffing system.

If −275 < *k*_1_ < −117, *k*_2_ = −0.1, *k*_3_ = −1, and *k*_4_ = −400, which satisfy (34), the eigenvalue equation of the equilibrium point is locally asymptotically stable. We choose  *k*_1_ = −200, *k*_2_ = −0.1, *k*_3_ = −1, and *k*_4_ = −400. The initial conditions of the master system 1 and the master system 2 are taken as *x*_1_(0) = 0.1, *x*_2_(0) = 0.2 and *y*_1_(0) = 0.2, *y*_2_(0) = 0.3, the initial conditions of the slave system 1 and the slave system 2 are taken as *X*_1_(0) = 0.3,  *X*_2_(0) = 0.4 and *Y*_1_(0) = 0.5, *Y*_2_(0) = 0.6, so the initial conditions of the error system are set to be *e*_1_(0) = 0.2, *e*_2_(0) = 0.2, *e*_3_(0) = 0.3, and *e*_4_(0) = 0.3. In Figures 3 and 4, we can see that all error variables have converged to zero; that is, we achieve the dual synchronization between the Van der Pol and the Duffing systems.

## 5. Conclusions

In this work, we construct a theory frame about dual synchronization of two different fractional-order chaotic systems and propose a method of dual synchronization. In addition, this method is used for designing a synchronization controller to achieve the dual synchronization of two different fractional-order chaotic systems. Finally, the proposed method is applied for dual synchronization of the Van der Pol-Willis systems and the Van der Pol-Duffing systems. The numerical simulations proves the accuracy of the theory.
